# Vaccine-masked spread of SARS-CoV2 in an elderly care home, and how to prevent a spill-over into the general population

**DOI:** 10.1007/s10389-021-01650-7

**Published:** 2021-09-14

**Authors:** Josef A. I. Weigl, Thorsten Werlang, Michael Wessendorf, Holger Helbing

**Affiliations:** Amt für Gesundheit Plön, Hamburgerstr.17/18 24306 Plön, Schleswig-Holstein, Germany

**Keywords:** Antigen test, Contact person, Ct value, Household, mRNA vaccine, Quarantine

## Abstract

**Aim:**

The vaccination campaign against SARS-CoV2 in Germany started at the peak of the second wave. An outbreak in an elderly care home occurred in our county at the time of the second vaccination. We describe a package of measures to control the outbreak and to prevent a spill over into the general population.

**Subjects and methods:**

After outbreak confirmation, a package of measures such as quarantine of the elderly care home, staff and visitors, and their households was implemented. By sequential testing, quarantine measures were lifted. Surveillance of staff and residents by rapid antigen test and symptom monitoring was used in parallel.

**Results:**

The outbreak was on-going for around 17 days until it was noticed by a symptomatic external staff member as index case. A total of 23 out of 96 residents (24.0%) and nine out of 114 staff (7.9%) were infected. Three residents died. Effective first-dose vaccine coverage was 85.4% in residents, 27.4% in internal, and 10.5% in external staff. Given the long latency period, the use of household quarantine prevented a spill over into the public. Already 16 days after notification of the index case the outbreak could be declared over.

**Conclusions:**

Interferences between vaccination coverage and outbreak characteristics in regard to an extended latency period were observed. Household quarantine of case as well as contact households is of increased importance in the era of vaccination to prevent further spread into the general population until population-based control measures and lockdowns can be lifted.

## Introduction

The turn of the year 2020 to 2021 was at the peak of the second wave of the current pandemic in our region (Robert Koch-Institute 2020). At the time community transmission and extensive outbreaks in the regional hospitals were on-going. The UK variant (B 1.1.7) was spreading and spill-overs into the broader community with enhanced transmission were imminent. After implementing vaccination centres to be ready for service as of 15 December 2020, vaccination started in our region (Schleswig-Holstein) on 27 December 2020. Many outbreaks in elderly care homes (ECHs) were notified before, during, and immediately after the immunisation visits by teams of the Federation of Managed Care Physicians, the Kassenärztliche Vereinigung (KV).

### Outbreak detection

On Tuesday 16 February 2021 our department, the County Health Department Ploen (CHD Ploen), was notified by a neighbouring CHD in regard to a PCR-positive-tested external staff member of an elderly care home (ECH) in our county. This index case had had respiratory symptoms since 11 February 2021 and was on duty until then. An antigen rapid test was negative, but a consecutive PCR in the top regional laboratory was positive, with a ct value of 20 (ct above 30 was considered non-contagious). The exploratory interview revealed no suspicious contacts outside the job. We were reassured that a FFP2 mask was worn during work, and the contacts to the residents were below 15 min each.

With this paper, we want to illustrate the containment tactics of our response. The emphasis is on the configuration of measures taken, from which we hope others can profit. The ultimate goal was to control the outbreak in the ECH as well as to prevent a spill-over into the general population as swiftly and effectively as possible.

## Subjects and methods

### Study design

The classical principles of an outbreak investigation were used (Goodman et al. 1996). After confirmation of the outbreak, the immediate next steps and measures are described. After a comprehensive regimen of quarantine measures, a de-escalating and fine-tuning tactic by stratification of measures according to laboratory results and households was used.

### Case definition

A case was either a resident or an internal or external staff member of the ECH, initially as of 4 February and later corrected to the set point 30 January 2021 with PCR confirmation of an SARS-CoV2 infection. An epidemiological curve is not shown, since the incidence of cases was dependent on the timing of the swabbing actions.

### Laboratory analysis

The ECH used the Siemens (Clinitest®) rapid antigen test. All samples of the mass swabbing actions were investigated in our preferred laboratory using a N-gene and E-gene based real-time RT-PCR. All positive samples were screened for the N501Y mutation and the delH69/V70 deletion by a specific S-gene based PCR. Solitary samples taken by individually caring physicians were tested also in other laboratories.

### Statistical analyses

Only absolute numbers, percentages, and proportions are used, stratified by resident and staff (internal and external) and according to residential zone and vaccination status.

### Ethical statement

Since our CHD was the primary public health institution responsible for this particular ECH, there are no constraints on the actions taken apart from the general principles of the applying German laws, especially the Infectious Diseases Control Act of the year 2001, latest modification 2020.

## Results

The ECH consisted of three residential zones and the option for short-term care, with a total of 96 residents, mean age 84 years [95% CI (81.8; 85.7), range 54–101] and 114 staff, mean age 46 years [95% CI (43.3; 47.8), range 19–67], on 30 January 2021: 95 internal and six external care staff and 13 external therapists (Table 1). The staff is not described according to zones, since many staff members were active across zones. A first vaccination visit took place on 8 January and a second on 6 February, i.e., after/at possible exposure to the wild virus after introduction into the ECH.

**Table 1:** Overview

Subjects on 30 January 2021	Residents: *n* = 96 (100%)	Staff total: *n* = 114 (100%)	Staff internal: *n* = 95 (100%)	Staff external: *n* = 19 (100%)
Location				
Zone 1	31 (32.3)			
Zone 2	31 (32.3)			
Zone 3	29 (30.2)			
Short-term care	2 (2.1)			
Vaccination status (shots)				
None	9 (9.4)	68 (59.6)	51 (53.7)	17 (89.5)
One	6 (6.3)	20 (17.5)	20 (21.1)*	0
Two	81 (84.4)	26 (22.8)	24 (25.3)	2 (10.5)

* immunisation not yet effective since 18 out of 20 vaccinated on 6 February (second vaccination visit)

### Outbreak confirmation and staging

On the morning of Wednesday 17 February, the day after notification, all residents and those staff members who were present were tested with the rapid antigen test (Clinitest®, Siemens) under control of the management of the ECH. Twelve residents and one staff member tested positive. In the afternoon, a team of our CHD took swabs of all antigen-test-positive persons and two residents who had had onset of symptoms in the meantime. Further exploration and interviews with the management, staff members, and residents were carried out at the same time.

On Thursday 18 February morning, ten of the 12 residents with positive antigen tests, the one staff member, and the two symptomatic residents were confirmed as SARS-CoV2-positive by PCR. All antigen-test-positive persons had ct values below 26. This was proof that an outbreak was on-going, and a comprehensive testing of all the 95 internal and the remaining four external (of a total of six) staff members, whether on duty or not, was planned for the next day. The staff members not on duty during the entire period were excluded. External “staff members” such as therapeutic specialists were instructed to get a PCR test at their closest facility, if eligible due to exposure. Swabs of the residents for PCR testing were postponed for 1 day, since they were confined to the institution.

On Friday 19 February, swabs from 87 available personnel were taken by our team. An additional six staff members were PCR-confirmed, adding up to eight total to that date (Table 2).

**Table 2:** Attack rates stratified by residents and staff

Subjects on 30 January 2021: subgroup	Residents: *n* = 96 (100%)	Staff total: *n* = 114 (100%)	Staff internal: *n* = 95 (100%)	Staff external: *n* = 19 (100%)
Symptom positive initially	3 (3.1)	3 (2.6)	2	1
Symptom positive totally	5 (5.2)	4 (3.5)	3	1
Hospital admissions	1 (1.0)	0	0	0
Deaths	3 (3.1)	0	0	0
PCR-positive initially**	22 (22.9)	8 (7.0)	6	2
PCR-positive later	1 (1.0)	1 (0.9)	0	1
PCR-positive totally	23 (24.0)	9 (7.9)	6	3
PCR-positive at end***	1 (1.0)	0	0	0
PCR-positive total and vaccination status				
None	5	7	4	3
One	1	2*	2*	0
Two	17	0	0	0
PCR-positive and ct values per residential zone		(ct 12–26)		
Zone 1(*n* = 31)	3 (ct 18–21)	n/a	n/a	n/a
Zone 2 (*n* = 31)	10 (ct 14–35)	n/a	n/a	n/a
Zone 3 (*n* = 29)	9 (ct 18–28)	n/a	n/a	n/a
Short-term care (*n* = 2)	1 (ct 22)	n/a	n/a	n/a

* immunisation not yet effective since 18 out of 20 vaccinated on 6 February 2021 (second vaccination visit)

** at initial outbreak staging (17 to 21 February 2021)

*** at end of outbreak (3 March 2021)

n/a: not applicable

On Saturday 20 February, all not yet PCR-tested residents plus seven further staff members not available on the previous day were PCR tested by the KV team. Ten additional residents, particularly those with higher ct values, were found positive, adding up to 22 positive residents (22.9%) and eight staff (7.0%). In zone 2, three residents had the highest ct values (31, 33, and 35 respectively) and therefore the most advanced infection. No variants of concern were diagnosed in any of the positive cases. Additional interviews based on these findings led to the primary case, a lady with a ct value of 31 who was in the local university hospital the entire day of 27 January, in the middle of an on-going outbreak there, and most probably acquired the infection there. No better alternative source could be identified. She had no vaccination so far, showed no symptoms and had contact to her peers in the same zone (zone 2, the hot zone) (Table 2). Given this new information, the set point for the case definition was reset to 30 January, the day of earliest transmission from the supposed primary case within the ECH. From zone 2, it was supposed that the infection was spread by junior staff and external staff to the other zones. An introduction of the infection by the external vaccination team on 6 February could be excluded based on the testing and vaccination history.

### Outbreak control measures

Immediately on Thursday 18 February, the morning when the outbreak was confirmed by the incoming PCR results, comprehensive measures were taken. The entire ECH was put under quarantine and visitors only allowed for moribund residents, or a single visitor across the coming fortnight for psychologically impaired residents. Visitors after 30 January and their families were put into quarantine, the so-called quarantine of contacts’ households (HhQ2°). For all but one family, it was possible to lift quarantine already on 21 February, the day of the PCR results of the residents.

All PCR-test-negative staff members were put under quarantine, but were allowed to work to keep the ECH functional based of the premise that an antigen test is performed daily before starting duty on top of personal protective equipment (PPE). In the local jargon we call this “tunnel-quarantine”. Staff members could come to work by private transport or by the ECH shuttle, but were prohibited to use public transport. Their family members were put under quarantine, too (HhQ2°). When the swabs were taken on Friday 19 February by our own team, staff members could state on a questionnaire whether they could separate themselves from their families at home from now onwards until the end of the quarantine period in case the PCR result was negative. If so, the quarantine of their family members was lifted. A junior staff member turned out to be positive, and his entire family, who were under quarantine already by this measure since 18 February, tested PCR-positive. So did one other co-worker and a family member. A spill over in the population was prevented to the best of our knowledge. The time interval from 18 February to 20 February protected by HhQ2° was of key relevance to prevent further spread from already incubating or shedding family members into the community, given the long exposure history as of 30 January, i.e., three serial intervals.

### On-going surveillance and maximum attack rate

In agreement with the management of the ECH, the following measures were put in place for surveillance: daily antigen-testing of the three shifts before work and notification to the CHD Ploen, notification of symptom onset in residents and staff members, and notification of hospitalisations and deaths. One staff member was confirmed positive 3 days after the last swabbing by his own physician, and one resident showed symptoms and was confirmed positive, adding up to a total attack rate of 23 out of 96 residents (24.0%) and nine out of 114 staff (7.9%). One resident of those vaccinated was admitted to hospital. Three residents died; two after two doses of vaccination, and one who refused vaccination in his final stage of cancer.

On 3 March, another comprehensive PCR-test action was performed for residents and staff, again by the team of the KV, and all PCRs were negative except one for a resident with an original ct value of 12, who now had a ct value of 27; her isolation was extended for one further week.

### Vaccination status and vaccine efficacy

With 84.4% in residents and 25.3% and 10.5% for permanent and external staff respectively, vaccination coverage was very heterogeneous. This is also reflected in the attack rate per vaccination status (Table 2). It is important to note that all staff members except one with one vaccination only obtained their shot on 6 February, and thus had no time to build up their immune response and could be considered unvaccinated. The two doses in residents and staff in fact only warranted a one-dose situation, since the second dose was unlikely effective at the time of exposure. A second dose given at the second visit could not yet guarantee full protection by a two-dose regimen at the time of exposure, in spite of a faster booster reaction by a second dose.

## Discussion

Ploen County on the Baltic Sea shore is the county with the lowest cumulative incidence of SARS-CoV2 across the entire pandemic in Germany so far (Tagesspiegel 2020). Therefore, a strong effort was made to prevent a spill-over into the general population. Since exposure of the index case (an external staff member) was most likely within the ECH, exploratory and low threshold testing of all residents and available staff by rapid antigen test was performed and turned out a positive signal. This signal could be confirmed by a fast first swabbing action by our own team with confirmation of an outbreak within the ECH by PCR. Just 10 days before the index case occurred, the ECH had its second vaccination visit.

With confirmation of the outbreak, a comprehensive pattern of quarantine measures was launched according to the principle “hit it hard and early”. From there, a de-escalating tactic was followed according to the diagnostic work-up and staging of the outbreak. Putting an entire ECH under quarantine and not allowing visitors into the facility is a standard principle. Also the option of a “tunnel-quarantine” for staff members is widely practiced, given the shortage of workers in the elderly care sector and the necessity for care by staff, which is familiar to those fragile elderly people with a considerable fraction of residents with dementia. To order immediate household quarantine for the households of contact persons (HhQ2°), however, is less commonly practiced, but a highly efficient tool (Weigl 2021). The burden for most households, whether the households of employees or visitors, could be lifted quickly (here 3 to 4 days) in case a reliable separation within the household could be adhered to and the contact person could be proven PCR-test-negative at the time of separation or where the potential source person was proven PCR negative. Given the long-ago-exposure set point of 30 January, with around three cycles of viral spread having taken place according to the generation time of around 6 days (He et al. 2020) and the problem of transmission by asymptomatic persons, the threat of a spill-over into the general population via household members, in this case the households predominantly of the staff members, was real.

The number of cycles of viral spread explains the considerable size of the outbreak with 32 persons in total, 24.0% (*n* = 23) of the residents and 7.9% (*n* = 9) of the staff; according to a recent statistics from the Robert Koch-Institute an at least upper-mid-size outbreak (Schweickert et al. 2021). One very important reason why it took so long to notice the outbreak is that it took an unvaccinated staff member working under PPE becoming infected and revealing symptoms. The reason why no resident so far had showed up with symptoms might well have been a considerable proportion of residents with a sufficient degree of immunity by the first vaccination visit of 8 January. The vaccination status of an ECH can well increase the latency period until an outbreak is noticed. However, the observation of spreading by asymptomatic residents and staff in ECHs had also already been made early in this pandemic, long before the vaccination era (Leister 2020).

At this moment, a high incidence of outbreaks in ECHs settings occurred in our region. On many occasions, suspicion was raised that there might be a connection to the vaccine (mainly Comirnaty®) or the vaccination teams. The former can be excluded, since it is an mRNA vaccine and not a life-attenuated vaccine; and the latter could be excluded, since vaccination teams are closely monitored by PCR-testing, as was the case here. The explanation for the phenomenon of many outbreaks at the time of vaccination is that the local vaccination campaign for the ECHs started at the peak of the second wave and therefore was a coincidence. The death toll of 3.1% of the residents (*n* = 3, of whom two were vaccinated) is plausible and at the lower margin reported (Schweickert et al. 2021), given the proven vaccine efficacy of at least 52% early after the first dose (Pollack et al. 2020). Given the exposure time and the time of the second vaccination visit, a booster response by the vaccine was unlikely for many residents. A booster was, however, rendered by the wild-type infection, at least in those in whom an infection could be documented. In the long run, this could even be an advantage given the broader immune response from a wild-type infection (Weigl et al. 2021). Whether the death toll would have been greater without vaccination can be assumed but not proven. The attack rate was higher in non-vaccinated residents (five out of 14 unvaccinated and 6 February first vaccinated) than in at least once vaccinated (17 out of 81 twice and one once vaccinated), which shows that the virus will find the unvaccinated even in a setting with an high vaccine coverage. Vaccine coverage was higher in the internal staff members than in external ones, which demonstrated that all present staff whether external or internal, whether care personnel or technical staff, have to be vaccinated to lower vulnerability to the function of the ECH and personal risk. Not all staff members were ready for vaccination at the first visit, but many decided to participate in the second visit. This means they had their priming vaccination at 6 February, and given the necessary time window to build up a proper immune response (Pollack et al. 2020), they can be assumed naïve at the time of exposure. None of the 24 staff members who were vaccinated already at 8 January tested positive for SARS-CoV2, which is remarkable given that they can be counted as only vaccinated once. It was also remarkable for us that at the follow-up testing on 3 March already all but one formerly PCR-positive persons had turned negative, which hints at a faster viral clearance in vaccinated and primed persons, although elderly. On the other hand, the sequence of events proves that SARS-CoV2 mRNA vaccines are non-sterilising, i.e. vaccinated persons can transmit the virus. However, the caveat of a not-yet-matured booster response due to the timeline has to be mentioned here.

The so-called “tunnel-quarantine” in our local experience is a valid tool for maintaining the function of the ECH; augmented by surveillance by daily rapid-antigen testing and symptom monitoring after a baseline PCR-negative test, it increased the certainty that no further infections were imported into the facility. It is important to notice that this was done before the new testing strategy in Germany, with routine antigen testing in the general population and workforce, became effective. One staff member on sick leave was tested positive outside and one further resident got symptoms and tested PCR-positive.

The outbreak could be declared as ended already as of 4 March, which was surprisingly fast, and we were sufficiently sure that no spill-over into the population did occur.

### The role of the laboratory

The ct values from a competent laboratory are of great value, as could be shown here. The high ct values in three of the zone 2 residents led the suspicion to one of the three ladies with the high ct values. To this end, the ct values were essential to identify the source, the primary case. It is awkward that to this date laboratories in Germany cannot be obliged to communicate the ct values or the viral load to the CHDs routinely. We consider all excuses made as not valid. On the contrary, they are more suited to covering up insufficiencies in the laboratories than shortcomings of the value itself. All diagnostic results in medicine have to be appraised critically by the user. Ct values and the reiterative interview in the setting were essential for success in disclosing the sequence of events in the outbreak reported here.

The rapid antigen test fulfilled its mission, since by this tool the outbreak signal was identified. At the same time, it cannot be used to stage the outbreak precisely, since its sensitivity and specificity are not sufficient for this purpose. Once a person is tested negative by PCR at baseline, antigen tests again can help in the on-going surveillance within staff and residents, since any new case would need to pass through the time window when ct values would be low enough for the antigen test to turn positive.

### The concept of the incubation period

There is a repeated need to remind hospitals and care facilities about the nature of the incubation period when admitting and re-admitting patients and residents respectively. Many times the error is made that a person is assumed not to be infected if a PCR test after possible exposure is negative, in analogy to the MRSA screening. Even with hospital doctors this is an issue. The right-skewed distribution of the incubation period comes on top (Lauer et al. 2020, Weigl 2020).

### Lessons learned

Vaccination efforts have to be maintained after the formal two visits by the KV team. Very often, external and technical staff members are not in focus for vaccination. The window of increased willingness to accept vaccination after an outbreak should be leveraged better. Vaccination should be offered as easily accessible as possible. In the near future, hopefully this issue will be resolved by primary care or workforce physicians involved in care within the ECHs.

Efficacy of isolation is guaranteed by swabbing activities and on-going monitoring. Efficacy of tracking is enhanced by household quarantine for case households as well as contact households (Fraser et al. 2004, Weigl et al. 2021). “Tunnel-quarantine” is an effective and safe way of maintaining the function of the institution. In the outbreak here, visitors did not play a pronounced role. However, a minimum of visits should continue to be allowed even in an outbreak, e.g. for moribund or psychiatric residents.

Communication within an outbreak situation is known to be a delicate task and we only want to mention on this occasion that there remained a discrepancy of view between our team and local politics in regard to when to disclose the name of the ECH to the media.

## Conclusions

After notification of the index case, fast action for outbreak confirmation and the necessary measures by the ECH management and the CHD were essential for success. Then detailed testing followed for fine tuning and de-escalation of measures according to the test findings.

With increasing vaccination coverage, outbreaks can be masked for a longer time period and the virus can spread more extensively than before in spite of a shorter and less intense shedding of the virus by vaccinees. In this way the virus will still find those indiviuals who are SARS-CoV2-naïve, e.g. the unvaccinated subjects. Household quarantine in case households as well as contact households is essential also in outbreaks to prevent a spill-over into the public, given the longer latency period in institutions with considerable vaccination coverage.

## Data Availability

All data are in the manuscript.

## Abbreviations

CHD: county health department
ct value: cycle threshold value
ECH: elderly care home
KV: Kassenärztliche Vereinigung
